# Legal Notes for Institutional Workers

**Published:** 1913-07-12

**Authors:** 


### LEGAL NOTES FOR INSTITUTIONAL WORKERS.

**(The Editor will be pleased to answer Legal queries in this column.)**

### Consent to Operation.

All things considered, there have been in this country amazingly few leading cases arising out of the knotty problems of consent to operation. United States law does not, of course, bind English Courts; but, inasmuch as the same general principles of law govern such cases there as here, a recent case in the New Jersey Supreme Court is of some interest. A surgeon undertook to perform a radical cure of a left inguinal hernia, and to do it gratuitously. When the patient was anæsthetised and ready for the operation to commence, a more serious rupture was found to exist on the right side. The surgeon accordingly dealt first with the right hernia, intending to cure the left one afterwards. However, by the time the first operation was complete, the patient's condition was not good, so the further operation was not commenced. The patient brought an action for assault, and was awarded substantial damages; but the higher Court set aside the decision on appeal. The grounds for this were that the use of anæsthesia modified the fundamental common-law rule that consent must be given before any operation is done. Anæsthesia has postponed to the period of relaxation and unconsciousness the making of the complete and final diagnosis of the case, by which time consent can no longer be asked or obtained. These changed conditions are all for the patient's benefit, and to allow them to be used as a basis of actions for assault or *mala praxis* would be contrary to the public interest. "When a person has selected a surgeon to operate on him, and has appointed no other person to represent him during the period of unconsciousness that constitutes a part of such operation, the law will by implication constitute such surgeon the representative of the patient and will . . . cast upon him the responsibility of so acting in the interests of the patient that the latter shall receive the full benefit of that professional judgment to which he is legally entitled." This decision is safeguarded by language which makes it safe from abuse, and confers no liberty upon the surgeon to carry out a more extensive or more dangerous operation than that for which he has obtained consent.

### A Difficult Point.

A very curious point—or series of points—has been raised by Dr. A. C. D. Firth in connection with a case of poisoning treated at the West London Hospital. Although the institution itself could hardly be held responsible for what occurred, its officers might possibly have been called upon for opinions and explanations had the course of events been slightly different in one respect. The facts are that a woman took acetate of lead in some quantity with the object of causing a miscarriage. In this she was successful, and she also succeeded in producing very serious lead poisoning. She was admitted to the West London Hospital, where these facts were discovered. The husband (a police-constable) was thereupon informed, and he promptly removed his wife, who was still very ill, from the hospital against the desire of the medical officers. She recovered; but had she not done so, a number of queries are suggested by Dr. Firth for legal experts to decide. First, could the husband have been indicted for manslaughter by wilful negligence? Or would he have been an accessory before the fact to his wife's suicide, although himself quite innocent of any complicity in the administration of the poison? Or would he be liable as an accessory after the fact, supposing some third person supplied and administered the poison? He concludes that probably the legal status conferred by the marriage bond would protect the husband, though we are not at all sure that this view is correct. As a further question we should like to propound this one: Could the hospital authorities or the hospital officer concerned be sued for damages by the patient, whose future domestic concerns might obviously be very seriously prejudiced by the information thus divulged? Unless a defence of privilege could be sustained, it is quite probable that an action might lie.

